# GENDER-SPECIFIC APPROACHES TO HEALTHY AGING WITH HIV

**DOI:** 10.1093/geroni/igz038.2809

**Published:** 2019-11-08

**Authors:** Tonya N Taylor, Ruth Finkelstein

**Affiliations:** 1 SUNY Downstate Medical Center, Brooklyn, New York, United States; 2 The Brookdale Center on Aging, Hunter College, New York City, United States

## Abstract

Older adults with HIV (OAH) evidence a significant burden of disease, characterized by high frequencies of non-HIV related comorbidities that result in multimorbidity and polypharmacy decades earlier than non-HIV infected older adults. Commonly observed comorbidities among OAH 50 years and older include cardiovascular disease, hypertension, diabetes, osteoporosis, and cancers. Geriatric conditions such as frailty, functional impairment, chronic inflammation, and cognitive dysfunction are also prevalent. Poverty, unemployment, housing, and food insecurity, as well as stigma-driven discrimination, persistent social isolation and high rates of depression, can make health-related challenges associated with aging overwhelming, resulting in a diminished capacity for self-care. Older men and women with HIV; however, are aging differently due to gender-specific differences in comorbidity profiles, polypharmacy burden, social networks, and intersectional stigmas (HIV/AIDS, ageism, racism, sexism, classism, homophobia, and transphobia). This symposium explores the impact of gender on healthy aging among OAH. Dr. Brennan-Ing and colleagues describe specific strategies for achieving healthy aging among female OAH in NYC and Oakland, CA. Dr. Rubtsova and colleagues explore multiple meanings of “successful aging” among older women in Atlanta and Brooklyn, and the role of resilience that empowers them as long-term HIV survivors. Dr. Taylor qualitatively examines the impact of biopsychosocial factors on notions of “Healthy HIV Aging” among older gay, bisexual and heterosexual men with HIV. Dr. Ruth Finkelstein will discuss the public health implications for developing equitable and gender-specific and intersectional HIV prevention and wellness interventions and suggest future areas of inquiry to improve HIV outcomes across the care continuum.

